# Novel Mutations of the *Tetratricopeptide Repeat Domain 7A* Gene and Phenotype/Genotype Comparison

**DOI:** 10.3389/fimmu.2017.01066

**Published:** 2017-09-07

**Authors:** Reyin Lien, Yung-Feng Lin, Min-Wei Lai, Hui-Ying Weng, Ren-Chin Wu, Tang-Her Jaing, Jing-Long Huang, Shih-Feng Tsai, Wen-I Lee

**Affiliations:** ^1^Division of Neonatology, Department of Pediatrics, Chang Gung Memorial Hospital, Taoyuan, Taiwan; ^2^Institute of Molecular and Genomic Medicine, National Health Research Institutes, Zhunan, Taiwan; ^3^Division of Gastroenterology, Department of Pediatrics, Chang Gung Memorial Hospital, Taoyuan, Taiwan; ^4^VYM Genome Research Center, National Yang-Ming University, Taipei, Taiwan; ^5^Department of Pathology, Chang Gung Memorial Hospital, Chang Gung University, Taoyuan, Taiwan; ^6^Division of Allergy, Asthma and Rheumatology, Department of Pediatrics, Chang Gung Memorial Hospital, Taoyuan, Taiwan; ^7^Division of Hematology/Oncology, Department Pediatrics, Chang Gung Memorial Hospital, Taoyuan, Taiwan; ^8^Primary Immunodeficiency Care and Research (PICAR) Institute, Chang Gung Memorial Hospital, Chang Gung University College of Medicine, Taoyuan, Taiwan

**Keywords:** refractory diarrhea, *tetratricopeptide repeat domain 7A*, inflammatory bowel disease, multiple intestinal atresia, combined T and B immunodeficiency

## Abstract

The gastrointestinal tract contains the largest lymphoid organ to react with pathogenic microorganisms and suppress excess inflammation. Patients with primary immunodeficiency diseases (PIDs) can suffer from refractory diarrhea. In this study, we present two siblings who began to suffer from refractory diarrhea with a poor response to aggressive antibiotic and immunosuppressive treatment after surgical release of neonatal intestinal obstruction. Their lymphocyte proliferation was low, but superoxide production and IL-10 signaling were normal. Candidate genetic approach targeted to genes involved in PIDs with inflammatory bowel disease (IBD)-like manifestation was unrevealing. Whole-genome sequencing revealed novel heterozygous mutations Glu75Lys and nucleotide 520–521 CT deletion in the *tetratricopeptide repeat domain 7A* (*TTC7A*) gene. A Medline search identified 49 patients with *TTC7A* mutations, of whom 20 survived. Their phenotypes included both multiple intestinal atresia (MIA) and combined T and/or B immunodeficiency (CID) in 16, both IBD and CID in 14, isolated MIA in 8, MIA, IBD, and CID complex in 8, and isolated IBD in 3. Of these 98 mutant alleles over-through the coding region clustering on exon 2 (40 alleles), exon 7 (12 alleles), and exon 20 (10 alleles), 2 common hotspot mutations were c.211 G>A (p.E71K in exon 2) in 26 alleles and AAGT deletion in exon 7 (+3) in 10 alleles. Kaplan–Meier analysis showed that those with biallelic missense mutations (*p* = 0.0168), unaffected tetratricopeptide repeat domains (*p* = 0.0311), and developing autoimmune disorders (*p* = 0.001) had a relatively better prognosis. Hematopoietic stem cell transplantation (HSCT) restored immunity and seemed to decrease the frequency of infections; however, refractory diarrhea persisted. Clinical improvement was reported upon intestinal and liver transplantation in a child with CID and MIA of unknown genetic etiology. In conclusion, patients with *TTC7A* mutations presenting with the very early onset of refractory diarrhea had limit improvement by HSCT or/and tailored immunosuppressive therapy in the absence of suitable intestine donors. We suggest that MIA–CID–IBD disorder caused by *TTC7A* mutations should also be included in the PID classification of “immunodeficiencies affecting cellular and humoral immunity” to allow for prompt recognition and optimal treatment.

## Highlights

### What Is Already Known about This Topic?

Human *TTC7A* mutations mainly disrupt intestinal and thymus epithelia leading to multiple intestinal atresia-combined (T and B cell) immunodeficiency.

### What Does This Article Add to Our Knowledge?

In addition to the PID classification of “combined immunodeficiencies with associated or syndromic features,” the *TTC7A* gene should also be included as an “immunodeficiency affecting cellular and humoral immunity.” Patients with biallelic missense *TTC7A* mutations not involving tetratricopeptide repeat domains and developing autoimmune disorders have a relatively better prognosis.

### How Does This Study Impact Current Management Guidelines?

For patients with *TTC7A* mutations other than bi-missense mutations involving tetratricopeptide repeat domains, an advanced prenatal diagnosis and the lack of intestine donors suggest that termination would be relatively humane while the use of Rho A inhibitors remains hypothetical.

## Introduction

The gastrointestinal tract contains the largest lymphoid organ in the body. It plays an important role in cellular immunity through lymphocytes, macrophages, and dendritic cells after contact with pathogens (1, 2) to produce humoral immunity of sufficient antibodies for opsonization, neutralization, and complement activation (3, 4). If these immune responses and intestinal epithelia do not effectively cooperate and mutually balance, unwanted or excess reactions can injure the epithelia, mucosa, submucosa, and connective tissue, consequently leading to persistent diarrhea despite nothing *per os*, hydration, antibiotic, and immunosuppressive therapy (1–3).

Increasing evidence indicates that the early onset of chronic colitis, and especially inflammatory bowel disease (IBD), represents at least 2 of 10 warning signs of primary immunodeficiency diseases (PIDs) including “little effect to treatment,” “recurrent infections,” and/or “failure to gain weight or grow normally” (5). The genetic defects causing PIDs have been shown to elucidate the pathogenesis of refractory colitis and some cases of monogenetic IBD involving defective IL-12/23 (6–12) and IL-10 signaling (13–17), profound T cell defects (18–24), nicotinamide adenine dinucleotide phosphate oxidase anomalies (25, 26), anti-apoptotic signaling of X-linked inhibitor of apoptosis (27–30), and nuclear factor kappa B essential modulator (NEMO) transcription signaling (31). These findings offer a critical new insight into the pathogenesis of refractory colitis in patients with PIDs and suggest that it could be cured by hematopoietic stem cell transplantation (HSCT).

In 2013, the *tetratricopeptide repeat (TPR) domain 7A* (*TTC7A*) gene mutations were first identified by whole-exome sequencing (WES) in a French–Canadian family suffering from refractory IBD-like diarrhea (32). TTC7A consists of nine TPR domains that mediate protein–protein interactions and assemble multi-protein complexes to regulate cell cycle, transcription, and protein transport (33). *TTC7A* mutations dysregulate the distribution of α6-integrin and actin in the epithelial surface (34). Thus, the upside down bipolarity mainly displaces intestinal epithelia and thymus thymocytes leading to continuous apoptotic enteropathy and lymphocyte depletion (33, 34). Herein, we presented two siblings with refractory colitis who were referred to our Primary Immunodeficiency Care and Research institute where we identified novel *TTC7A* mutations for the first time in patients of Chinese ethnicity. To comprehensively review this rare disease entity that directly affects intestinal epithelia as well as cellular and humoral immunity, we further analyzed the phenotypes, genotypes, treatment, and prognosis from a PubMed search (32–41) to hopefully guide optimal management.

## Materials and Methods

### Case Reports

#### Patient 1

**Figure 1:**
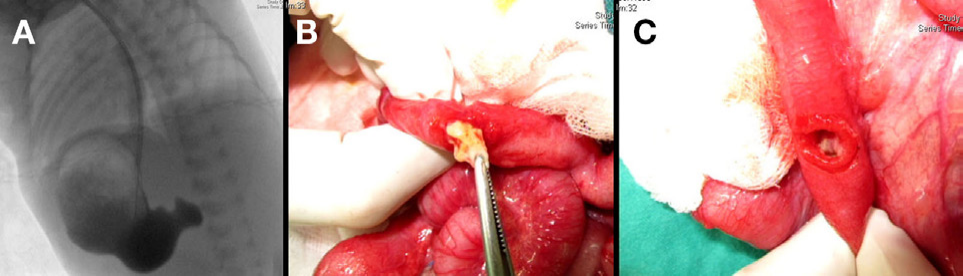
**(A)** Gastric outlet obstruction was identified in an upper gastrointestinal barium series as demonstrated during a surgical intervention showing **(B)** a grossly intraluminal nodule and **(C)** a duodenal web-like structure.

**Figure 2:**
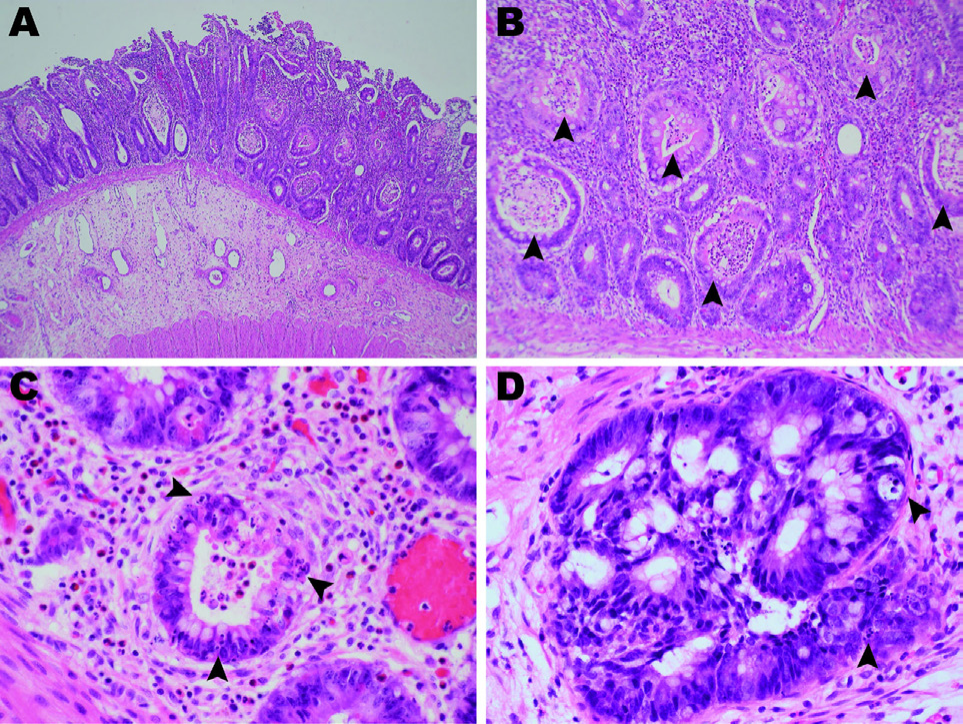
Photomicrographs of the ileum and colon (hematoxylin and eosin). **(A)** The terminal ileum of patient 1 showed erosion, severely inflamed mucosa, and a lack of Peyer’s patches (2× objective). **(B)** Widespread crypt abscesses (arrowheads) were noted in association with crypt destruction (10× objective). **(C)** The lamina propria was infiltrated by neutrophils and eosinophils with few lymphocytes. Apoptotic bodies (arrowheads) were frequently found in the crypt epithelia (40× objective). **(D)** The colon mucosa of patient 2 showed marked crypt epithelial hyperplasia and frequent apoptosis (arrowheads).

This patient was born to non-consanguineous parents *via* cesarean section because of polyhydramnios and fetal omphalocele and was then transferred to our hospital. Omphalocele was repaired when she was 1 day old. Non-bilious and coffee-ground drainage was noted in her orogastric tube, and an upper gastrointestinal barium series revealed a gastric outlet obstruction (Figure 1A) caused by a duodenal web-like structure, which was resolved by surgical removal (Figures 1B,C) when she was 14 days old. She subsequently suffered from persistent bloody mucous IBD-like diarrhea up to 10 times per day. No bacterial, parasite, or viral pathogens were detected by culture and polymerase chain reaction amplification, including human immunodeficiency virus, *Giardia*, cryptosporidium, and *Clostridium difficile* toxin. Aggressive antibiotic (teicoplanin, ceftriaxone, and cefotaxime), immunosuppressive (methylprednisolone and cyclosporine), and intravenous immunoglobulin treatment failed to cure the persistent diarrhea. Colonoscopy demonstrated colon stenosis 5 cm from her anus. *Staphylococcus epidermidis* sepsis occurred twice from the central line for total parenteral nutrition (TPN) accompanied by hypoalbuminemia, hypogammaglobulinemia, and a large amount of bilious orogastric drainage (around 150 cm^3^ per day). When she was 2 months old, jejunostomy and ileostomy were performed to allow for intestinal feeding. Histopathologically, the ileum and colon mucosa showed multifocal erosions, crypt hyperplasia, crypt abscesses associated with destruction, increased crypt apoptosis, and an expanded lamina propria infiltrated with neutrophils and eosinophils. Notably, Peyer’s patches were absent and only a few lymphocytes were noted in the inflammatory infiltrates (Figures 2A–C). Tandem mass spectrometry showed normal plasma amino acid and urinary organic acid concentrations, acylcarnithine profiles, and total and free carnithine levels. In addition, serum levels of lactates, pyruvates, ketone bodies, zinc, iron, ferritin, total iron binding capacity, and vitamin B12 were all within normal ranges. Congenital disorders of glycosylation syndrome and cystic fibrosis were excluded. Neonate-onset IBD associated with PIDs was compatible with her phenotype, and the possibility of an intestinal transplantation was discussed if a suitable donor became available. However, she unfortunately died of *Escherichia coli* sepsis at 8 months of age while waiting for a transplantation.

#### Patient 2

**Figure 3:**
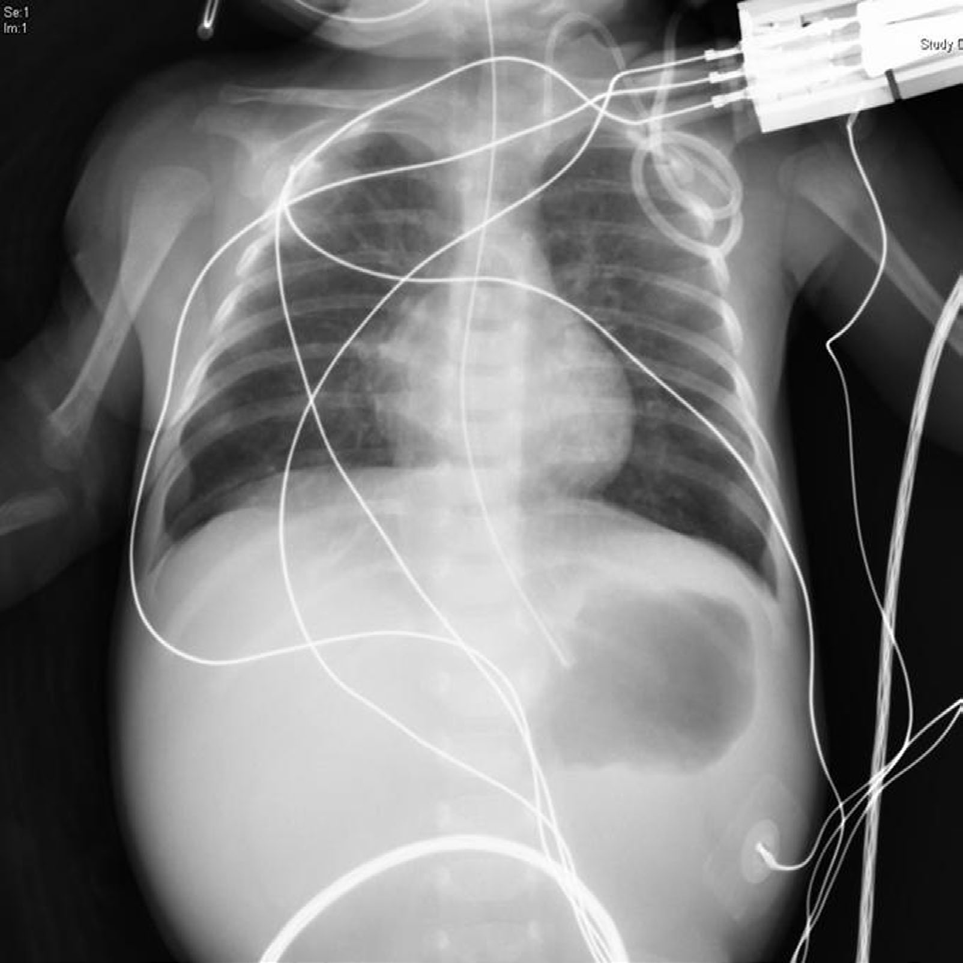
At 1 day of age, congenital pyloric atresia and narrow thymus shadow in her middle mediastinum were noted on a plain X-ray.

This patient was a younger sister of patient 1. She suffered from congenital pyloric atresia after birth (Figure 3). Although a gastroduodenostomy relieved her gastric outlet obstruction at 5 days of age, she subsequently developed bloody mucosal diarrhea over 10 times per day, which persisted despite nothing *per os*, TPN, aggressive antibiotic (imipenem), immunosuppressive, and anti-TNF-α biologic (0.5–1 mg/kg) treatment. Her severe diarrhea led to multiple perforations of the jejunum around 20 cm distal from the ligament of Treitz leading to septic shock at 2 months of age. Persistent abdominal distension, tachypnea, and tachycardia were noted after feeding. Central line-related *S. epidermidis* sepsis and disseminated intravascular coagulopathy occurred accompanied by a large amount of bilious orogastric drainage (around 150 cm^3^ per day), hyponatremia (111 meg/L), hypoalbuminemia, hypogammaglobulinemia, hyperlipidemia (triglycerol over 500 mg/dL), and convulsions. A colon biopsy revealed crypt hyperplasia, increased crypt apoptosis, and inflammatory infiltrates composed mainly of neutrophils and eosinophils (Figure 2D). Because of the similar presentation to her sibling, HSCT and intestinal transplantation were discussed if suitable donors became available. In addition to candidate genetic approach, we performed WGS to investigate genetic defects that could be checked in her next sibling during prenatal screening. Unfortunately, while preparing this manuscript, she succumbed to hepatorenal syndrome and metabolic distress from fulminant hepatic failure at 4 months of age.

### Basic Immunologic Function Assessments

In addition to infection, refractory colitis can be an autoimmune disorder in patients with PIDs, especially in those with combined T and B immunodeficiency (CID), Wiskott–Aldrich syndrome, chronic granulomatous diseases, defective IL-10 signaling, and hyper IgE syndromes. We first excluded well-defined immunodeficiency syndromes as demonstrated in patients with Wiskott–Aldrich syndrome by the size and number of platelets and its characteristics. We then evaluated lymphocyte proliferation, superoxide production, and IL-10 signaling.

The Chang Gung Human Investigation Committee approved all carried methods and all experimental protocols in this study, and the patients’ parents or guardians’ informed consent were obtained. We confirmed that all methods were performed in accordance with the relevant guidelines and standard regulations.

To induce lymphocyte proliferation, peripheral blood mononuclear cells (PBMCs; 10^5^/well) were incubated with different concentrations of phytohemagglutinin and pokeweed (PWM) mitogens for 3 days, or the *Candida* antigen and Bacillus Calmette–Guérin vaccine for 7 days. To measure reactive oxygen species (defined as H_2_O_2_ production), phorbol myristate acetate-stimulated (1 µM) polymorphonuclear cells (2 × 10^6^/mL) were incubated with the non-fluorescent compound 2′,7′-dichlorodihydrofluorescein diacetate dissolved in ethanol (Sigma, St. Louis, MO, USA), as previously described (42). To evaluate IL-10 signaling, purified PBMCs (5 × 10^6^/mL) were stimulated overnight with 50 ng/mL *E. coli* lipopolysaccharide (Sigma-Aldrich, St. Louis, MO, USA) alone or with 20 ng/mL recombinant human IL-10 (R&D Systems, Minneapolis, MN, USA). The supernatants were evaluated using a commercially available TNF-α enzyme-linked immunosorbent assay development kit in duplicate using a Tecan Sunrise enzyme-linked immunosorbent assay micro-plate reader (R&D Systems, Minneapolis, MN, USA).

The basic immunological levels, liver and renal functions of these two sibling patients are shown in Table 1. Quantitative analysis of their immunity revealed the lower borderline of naïve CD4 lymphocytes (CD4+CD45RA+) and memory B cells but higher expressions of CD8 memory cells and activated (CD3+HLA-DR+) T lymphocytes in patient 2 who had more severe IBD-like diarrhea at 3 months of age. Both had hypogammaglobulinemia (including lower IgG, IgA, and IgM by age) after 2 months, except for a higher IgE level possibly related to TPN infusion. Functionally, lymphocyte proliferation was lower; however, superoxide production from stimulated polymorphonuclear cells and IL-10 signaling in lipopolysaccharide-treated PBMCs were within the normal range, thereby excluding chronic granulomatous diseases and defective IL-10 signaling (TNF-α suppression).

**Table 1:** Hematogram and basic immunologic evaluations in our two female siblings.

	Patient 1		Patient 2	
Age (normal range)	1 day	2 months	7 days	3 months
WBC (6,000–17,500/mm^3^)	15,400	10,900	**↑ 24,800**	13,400
Segment (6,000–8,500/mm^3^)	**↑ 10,472**	7,848	**↑ 11,656**	**↑ 12,194**
Lymphocyte (2,000–13,500/mm^3^)	**↓ 1,848**	**↓ 872**	9,176	**↓ 268**
Hb (>10 mg/dL)	17.7	10.7	**↓ 9.6**	10.5
Platelet (>150/mm^3^)	231	**↓ 125**	563	**↓ 133**
AST (13–40 U/L)	42	23	**↑ 152**	46
ALT (<36 U/L)	9	6	**↑ 78**	36
Albumin (>3.5 mg/dL)	**↓ 2.8**		**↓ 2.9**	
BUN (5–20 mg/dL)	6.6	8.0	8.1	7.8
Cr (0.2–1.0 mg/dL)	0.5	0.1	0.5	0.1
CRP (<5 mg/dL)	<5	**↑ 36.1**	**↑ 159**	
**Lymphocyte subset percentage**				
CD3 (51–84)		60.1	80.3	94.8
CD4 (31–56)		32.5	33.3	16.0
CD8 (12–35)		23,7	48.9	60.3
CD19 (6–27)		20.8	15.2	**↓ 2.94**
CD19CD27 B memory (0.5–2.4)		0.54		**↓ 0.04**
CD4CD45RA naïve T (12–45)		27.7	31.4	**↓ 10.3**
CD4CD45RO T memory (3–26)		5.4	12.2	5.6
CD8CD45RO T memory (1–11)		1.0		**↑ 35.4**
Activated CD3HLADR T cells (4–26)		4.2		**↑ 58.9**
Natural killer CD16CD56 cells (3–19)		18.2	3.1	**↓ 2.2**
**Immunoglobulin level**				
IgM (49–156 mg/dL)		**↓ <4.4**	62.7	**↓ 17**
IgG (334–1,230 mg/dL)		**↓ 41.8**	**↓ 87.6**	**↓ 179**
IgG2 (30–140 mg/dL)		**↓ <30**		50
IgA (15–113 mg/dL)		**↓ <7**	39	**↓ <7**
IgE (<100 IU/mL)		<17.8	**↑ 2,920**	<17.8
**Lymphocyte proliferation (cpm)**				
PHA 2.5 μg/mL (29,228–58,457)		**↓ 1,453**		**↓ 4,928**
PWM 0.1 μg/mL (11,395–28,487)		**↓ 1,787**		**↓ 5,291**
*Candida* 2.5 μg/mL (5,351–13,328)		**↓ 532**		**↓ 142**
BCG 0.002 μg/mL (1,740–4,352)		**↓ 160**		**↓ 123**
**Superoxide production in PMN**				
(88–99%)		94.5		87.4
**TNF-α suppression in IL-10 signaling**				
(4.7–21.4%)		8.6		9.7

*PHA, phytohemagglutinin; cpm, counts per minute; **↑**, higher than normal ranges; **⤓**, lower than normal ranges*.

### Candidate Gene Approach

The candidate genes of CID prone to refractory diarrhea including IL-2 receptor common gamma chain (*IL2RG*), *ZAP70, CD3 gamma, ORAI-1* and *ITK*, and those also associated with refractory IBD-like colitis such as *FOXP3, STAT3, IL10, IL10RA, IL10RB, NEMO*, and *STAT1* were all sequenced (6–21, 43, 44). Every two oligonucleotide primers were selected to cover the entire coding region. These primer sequences were based on human genome sequences and are available on the Resource of Asian Primary Immunodeficiency Diseases website (45) or upon request.

### Whole-Genome Sequencing

Because of the wild types of the candidate genes in the first sibling, WGS was performed for index patient 2 and her parents using an Illumina HiSeq 2000 system (Illumina Inc., San Diego, CA, USA). The data were processed using CLC Genomic Workbench 9.5.2 software (Qiagen). Briefly, paired-end reads were removed from low-quality bases (Q < 30, Phred scale) and aligned to a human reference genome (GRCh37/hg19). The mapping parameters were set to default except for mapping length, which was set to 0.9 and mapping similarity, which was also set to 0.9 and only allowed to read the mapped reference genome once. Variants were filtered based on sequencing coverage (removed if the depth was <10) and by comparisons with a common variant database (dbSNP version 138) (46–48).

To confirm the WGS results by Sanger sequencing, total RNA was extracted from the PBMCs or from established lymphoblastoid cell lines using TRIzol (Life Tech. Carlsbad, CA, USA), followed by reverse transcription polymerase chain reaction as previously described (49). The two pairs of primer sequences of the *TTC7A* gene were based on human genome sequences NM 001288951.1 and designed to cover the whole coding region by three pairs: (TTC7A-328F: CAC TTC TTG GCC GCA CCT TC, TTC7A-1401R: CTT CCT CGA TGT TGT CCT TG; TTC7A-1254F: AGT GAG GAG TGC TAC TGG AG, TTC7A-2320R: GGA GTC TGG AAG CCT TCT GG; TTC7A-2123F: CCT GGA TGT TGT CAA CAT GG, TTC7A-3150R: GCT GCA GAG GCG TTC ACT AA). Simultaneously, the genomic DNA in exon 2 (forward: CCT GAG CCA TTC ATG TAC TG; backward: ACT AGC CAT ACT CCA AGT GC; around 450 nucleotides) and exon 4 (forward: ACT GCT AAC AACC ACC GTC; backward: TAG CAC ACA GGT GAC CTA TG; around 400 nucleotides) was amplified and confirmed based on NT_022184.15.

### Statistics

To understand the whole disease course, we searched for all reported patients with *TTC7A* mutations in a Medline search. Survival curves were calculated using Kaplan–Meier analysis to compare their prognoses based on the phenotypes, genetic mutations, autoimmune disorders, and whether or not they received HSCT. The first follow-up day was defined as their birth date because the majority of the patients with *TTC7A* mutations presented with intestinal obstructions in the prenatal and neonatal stages. The last follow-up day and duration were defined according to those reported in the studies. We defined the multiple intestinal atresia (MIA) phenotype as multiple levels in the gastrointestinal tract from the stomach to anus, and the CID phenotype as T, B, or natural killer lymphopenia, decreased mitogen or antigen lymphoproliferation, or hypogammaglobulinemia. We defined the IBD-like phenotype as frequent diarrhea with more than seven episodes of unformed stools within 24 h for longer than 2 weeks requiring parenteral nutrition in spite of aggressive treatment. All analyses were performed using GraphPad Prism software, and a *p* value of <0.05 was defined as being statistically significant.

## Results

### Genetic Analysis

**Figure 4:**
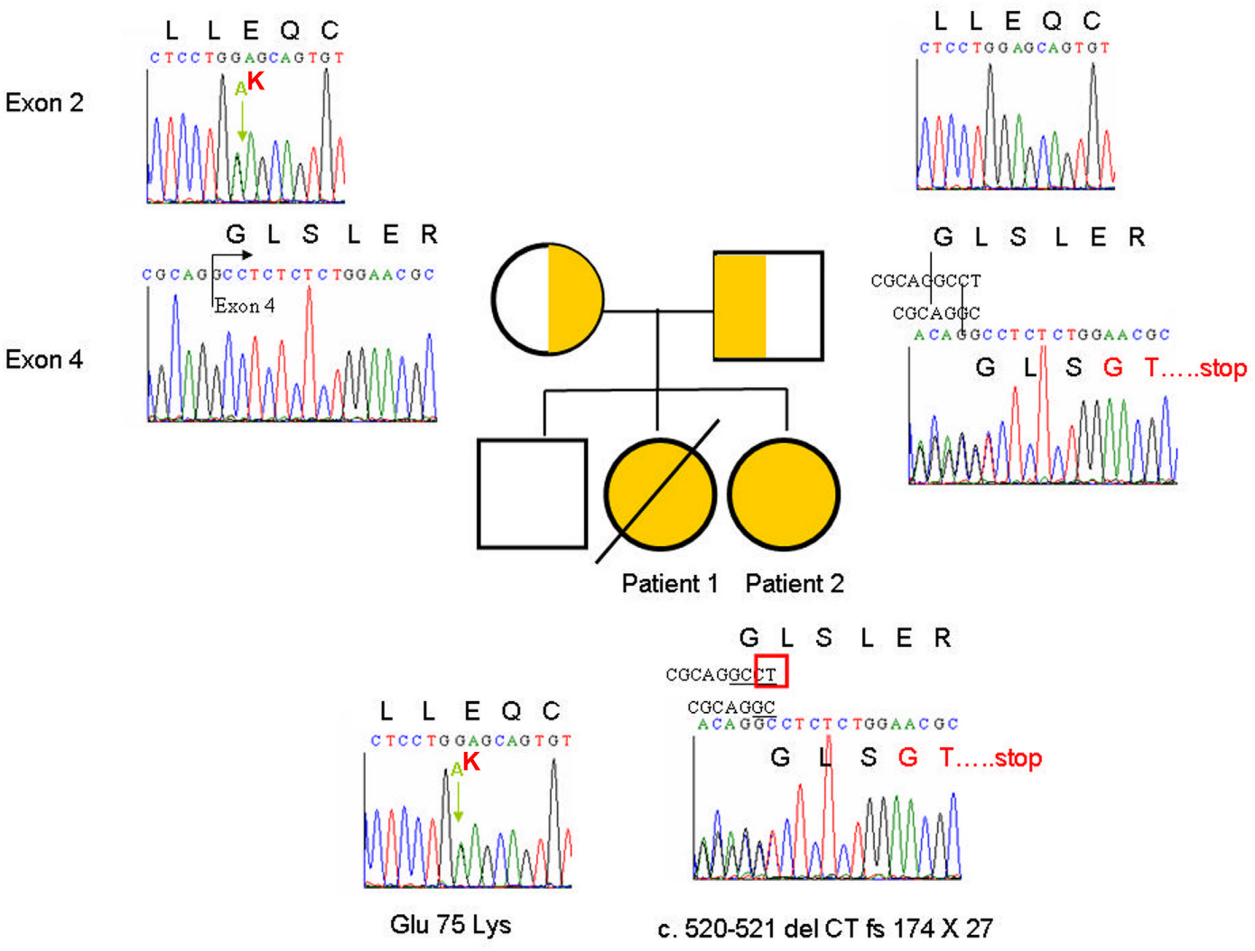
Sanger sequencing showed the heterozygous compound tetratricopeptide repeat domain 7A mutation: c.223 G>A in exon 2 causing Glu75Lys from the maternal allele and c.520-521 del CT fs174 × 27 in exon 4 from the paternal allele.

Patient 1 had a clinical diagnosis of CID characterized by lymphopenia, hypogammaglobulinemia, decreased lymphocyte proliferation, line-related infections, and refractory diarrhea. Candidate genes for PIDs were sequenced, but no mutations were identified at that time. WGS would have explained her genetic defect. Patient 2 had similar phenotypes. To identify disease-associated mutations, we performed WGS on genomic DNA from patient 2 and her parents. The results revealed that 10 genes may have contained compound heterozygous mutations that have not been reported in the dbSNP138 database and may affect protein function (Table S1 in Supplementary Material). Among them, TTC7A deficiency has been reported to result in IBD. To verify the mutations in the *TTC7A* gene, we applied Sanger sequencing on complementary and genomic DNA from the two sisters and their parents. The data showed that both patients had two *TTC7A* mutations on different alleles (Figure 4). The c.223 G>A mutation in exon 2 causing the Glu75Lys substitution was from the maternal allele, and the c.520-521 del CT mutation in exon 4 causing the fs174 × 27 frameshift, which resulted in the loss of all nine TPR domains was from the paternal allele (based on NM_001288951.1 and NT_022184.16).

### Comparison of Survival by Phenotype and Genotype

**Figure 5:**
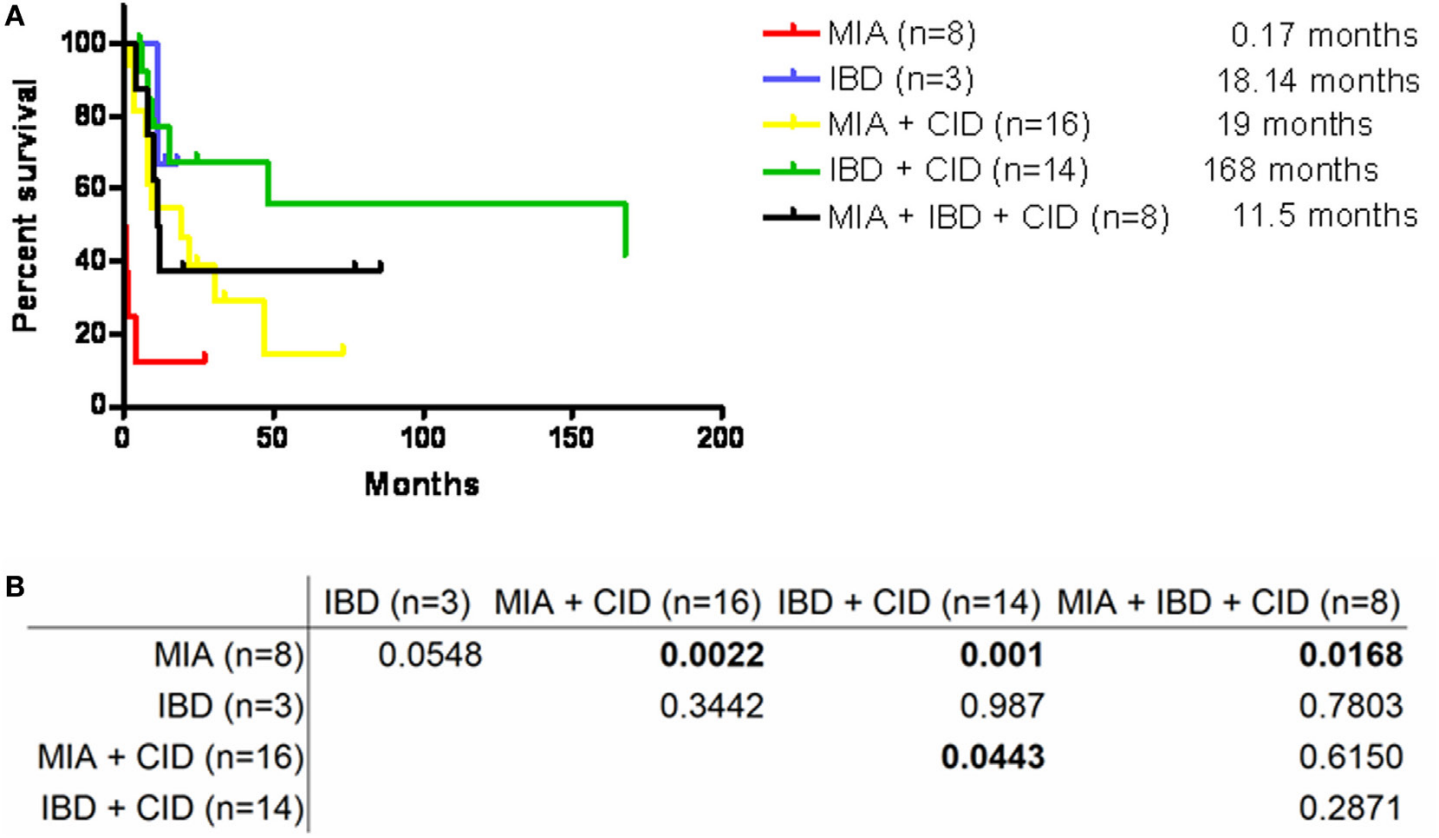
**(A)** Survival curve of the five phenotypes by Kaplan–Meier analysis showed **(B)** the significantly higher median lifespan in those with MIA + CID (median: 19 months), IBD + CID (168 months), and MIA + IBD + CID (11.5 months) compared to those with MIA alone (0.17 months); and also marginally significant with IBD + CID compared to MIA + CID. Abbreviations: MIA, multiple intestinal atresia; CID, combined T and B cell immunodeficiency; IBD, inflammatory bowel disease.

A PubMed search for the key words “intestine,” “atresia,” and “*TTC7A* mutation” excluding studies without genetic identification revealed 47 patients from 23 unrelated and 11 consanguineous families as well as our 2 patients from 1 family (Table S2 in Supplementary Material). A summary of the 5 kinds of presentations revealed MIA alone in 8 patients, IBD alone in 3 patients, the combination of MIA and CID in 16 patients, the combination of IBD and CID in 14 patients, and the complex phenotype of MAI, IBD, and CID in 8 patients. The median survival age of 5 days in the MIA alone phenotype was significantly shorter compared to the combined phenotypes (*p* = 0.0022 for MIA and CID; and *p* = 0.001 for IBD and CID) and the complex phenotype (*p* = 0.0168) (Figure 5). Sepsis, intestinal obstruction, and viral pneumonia were the three leading causes of mortality (Table 2). Notably, the patients with the combination of IBD and CID phenotype all had biallelic missense mutations, and those with other mutations had the isolated MIA, the complex and the majority of the combination of MIA and CID phenotypes.

**Table 2:** Mortality and mutation patterns associated with the five phenotypes in patients with TTC7A mutations.

Phenotype	Total	MIA + CID	IBD + CID	MIA	MIA + IBD + CID	IBD
Patient number	49	16	14	8	8	3
**Genotype**						
Both missense		2	14	0	0	2
Others		14	0	8	8	1
**Mortality cause**						
Sepsis	10	4	2	2	1	1
Intestinal obstruction	8	1	1	5	1	
Viral pneumonia	4	2			2	
HSCT	2		2			
Pulmonary emboli	1	1				
Pulmonary hemorrhage	1	1				
Cardiac arrest	1	1				
Gastric carcinoma	1		1			
Hepatic failure	1				1	
Cases of mortality	29	10	6	7	5	1

*MIA, multiple intestinal atresia; CID, combined T and B cell immunodeficiency; IBD, inflammatory bowel disease; HSCT, hematopoietic stem cell transplantation; TTC7A, tetratricopeptide repeat domain 7A*.

The 98 mutant alleles in the 49 patients expanded through the coding region and clustered on exon 2 (40 alleles), exon 7 (12 alleles), and exon 20 (10 alleles) including 47 missense, 32 deletion, 9 splicing, 9 nonsense, and 1 insertion mutation. There were two common hotspot mutations. The first was in a large consanguineous family and included 13 patients with c.211 G>A (E71K in exon 2) in 26 alleles who had the combined IBD and CID phenotype. The second came from a French–Canadian family with the founder effect of exon 7 (+3) AAGT deletion in 10 alleles that caused three kinds of phenotypes including the MIA alone phenotype in four homozygous patients, the MIA and CID phenotype, and the complex of MIA, CID and IBD phenotype in one heterozygous patient each.

With regards to the relative frequency of the 34 family cases rather than 49 individuals, there were 31 deletion, 20 missense, 9 nonsense, 7 splicing, and 1 insertion mutation, similarly concentrated on exon 2 (15 alleles), exon 7 (12 alleles), and exon 20 (10 alleles). As well as the founder effect of exon 7 (+3) AAGT deletion in 10 alleles, the 313-6 TATC deletion of exon 2 in 8 alleles replaced E71K in a large consanguineous family to become the most two common hotspot mutations.

**Figure 6:**
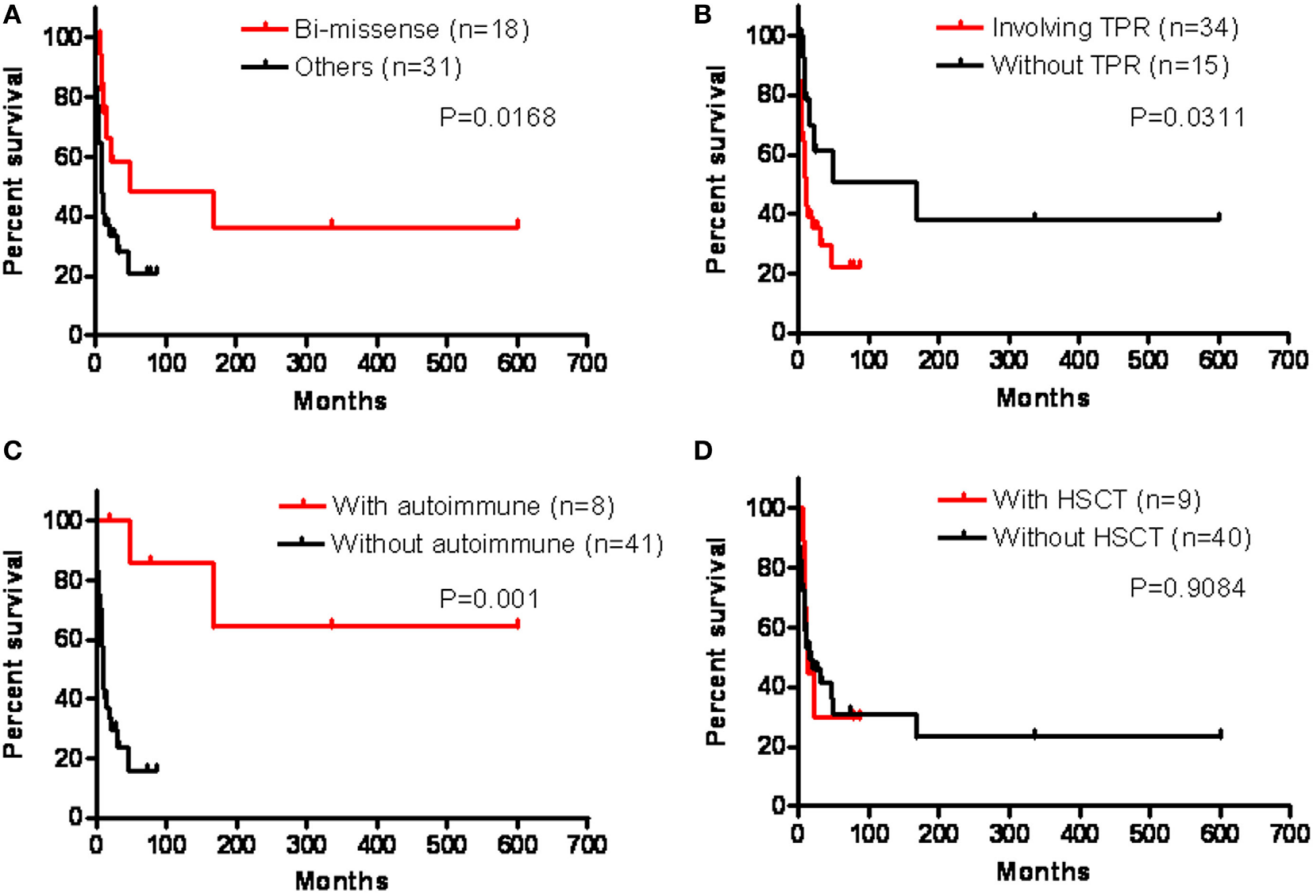
We analyzed their prognosis in the presence of genetic bi-missense mutations **(A)**, tetratricopeptide repeat (TPR) domain involvement **(B)**, autoimmune disorders **(C)**, and hematopoietic stem cell transplantation (HSCT) **(D)** by Kaplan–Meier analysis, which revealed a significantly better prognosis in those with bi-missense mutations (*p* = 0.0168), without TPR domains (*p* = 0.0311), and with autoimmune disorders (*p* = 0.001), but not significantly in those receiving HSCT (*p* = 0.9084).

Overall, the patients with biallelic missense mutations (*p* = 0.0168), unaffected TPR domains (*p* = 0.0311), and developing autoimmune disorders (*p* = 0.001) had a relatively better prognosis than those without (Figure 6). Although successful HSCT restored immunity to decrease the frequency of infection, improvements in refractory diarrhea were limited and the survival rate was still low in those receiving HSCT, and not significantly better compared to those who did not receive HSCT (*p* = 0.9084).

## Discussion

*Tetratricopeptide repeat domain 7A* mutations were first identified by WES in a French–Canadian family in 2013, who had the founder effect of four nucleotide deletions leading to skipping exon 7 and losing seven TPR domains (32). Since then, the two female siblings in the current study plus a few patients from Caucasian (35), Saudi Arabia (36), Norway (34), Sri Lanka (34), Israel (37), Serbia (36), Bosnia (36), Italy (36), Malaysia (38), Turkey (39), and Britain (40) have been reported to have *TTC7A* mutations causing the complex phenotype of MIA–CID–IBD disorders. Of note, approximately 75% of the patients (37/49) had the CID phenotype, which was mainly caused by a disorganized thymus prohibiting lymphocyte development, thus causing CID (combined B and T cell deficiencies in 27 and T cell deficiency in 10 patients). In addition to “combined immunodeficiencies with associated or syndromic features” in the updated PIDs classification (50), we suggest that the *TTC7A* gene also be categorized as an “immunodeficiency affecting cellular and humoral immunity” and be included in target exome sequencing for neonatal IBD-like refractory diarrhea to raise general awareness and prompt an early diagnosis.

In practice, treatment depends on which phenotype is present. Surgical resection is the main strategy to relieve single and multiple intestinal obstructions. After relieving the obstruction, refractory diarrhea underlying apoptotic enteropathy occurs, persists, and barely responds to immunosuppressive treatments including steroids, azathioprine, methotrexate, cyclosporine, sirolimus, tacrolimus, and anti-TNF-α agonists (infliximab and adalimumab) (40, 51), as seen in our patients. In addition to the comorbidity of CID, defective intestinal barriers and long-term central TPN lines increase infection susceptibility to the gut Gram-negative and cutaneous Gram-positive pathogens, which are slightly different from common opportunistic pathogens in patients with classic CID. Successful HSCT can restore immunity to overcome thymus dysfunction (40). However, the severity of intestinal inflammation and the frequency of stenosis underlying persistent apoptotic enteropathy can only marginally attenuate flare-ups rather than completely cure them (40). Superior to HSCT, a combined liver and small intestine transplantation rescued an 8-month-old boy, without identified genetic defects at that time, who had TPN-related liver cirrhosis and the combined MIA and CID phenotype (52). Interestingly, the recovery of most CD3+ and 50% of the CD19+ lymphocytes was attributed to donor intestine–intraepithelial lymphocytes, whereas the granulocytes and monocytes remained of recipient origin. His liver function, intestinal motility, and enteral feeding were normal 2 years posttransplant.

In micro-pathogenesis, *TTC7A* mutations directly reduced the transport of phosphatidylinositol 4-IIIα (PI4KIIIα), a major TTC7A-interacting protein, into plasma membrane for membranous stabilization. Indirectly, *TTC7A* mutations were not able to counter-balance Rho A activation for adequate actin-cytoskeleton polymerization. A study on 3-dimensional intestinal organoid cultures reported that α6-integrin transported by TTC7A–PI4KIIIα was normally expressed on the basolateral surface but aberrantly detected inside cell aggregation in patients with *TTC7A* mutations whose actin was also dislocated at peripheral regions but not normally expressed at the apical brush border facing the lumen in healthy controls (34). A Rho A inhibitor (Y-27632) *in vitro* has also been demonstrated to be able to potentially reverse disturbed intestinal epithelia and thymic thymocytes (33, 53), and large-scale clinical trials are warranted to elucidate these effects.

Three patients who had the homozygous E71K missense hypomorphic mutation without TPR domain involvement grew to old age and presented with intermittent IBD episodes (54). They tended to develop autoimmune disorders including alopecia, hemolysis, diabetes, thyroiditis, psoriasis, and onychomycosis as they grew older even though they mostly had lymphopenia, hypogammaglobulinemia, T, B, and natural killer deficiencies (54). In our analysis, the best survival rate was with the combined IBD and CID phenotype (median survival age 168 months), which was to some extent ascribed to these three patients who were reported to still be alive at 14, 28, and 50 years of age.

There are some limitations to this study. First, donor intestines are scarce, and to date, no intestine has been transplanted into a patient with identified *TTC7A* mutations. Among the nine patients receiving HSCT, three survived but suffered from persistent refractory diarrhea. Second, the intra-family patients with the same mutations could have diverse phenotypes. In the most extreme example with homozygous E71K mutations in the same family, some members died in the process of HSCT at 6 months of age, whereas one grew to 50 years of age. Further studies are needed to determine and recognize whether modified mechanisms can compensate defective TTC7A signaling to open alternative therapeutic avenues. Third, it is still challenging in multidiscipline centers to optimize the timing of intestinal transplantations and HSCT. More experience is required to reach a treatment consensus.

In conclusion, WGS or target gene panel sequencing for neonatal- or infantile-onset MIA–CID–IBD-like diarrhea can reveal the causative genetic mutations of *TTC7A*. The majority of patients with *TTC7A* mutations experience prenatal polyhydramnios and refractory diarrhea following surgical removal of the intestinal atresia, which continues to worsen in spite of immunosuppressive and biologic treatments and even successful HSCT to overcome T and B immunodeficiency. Although suitable intestinal transplantation can potentially eradicate recurrent MIA and IBD and also restore immunity from the donor intestinal lymphocytes, the scarcity of donors and the availability of an advanced prenatal diagnosis suggest that legal termination for those with *TTC7A* mutations other than bi-missense mutations involving TPR domains is a relatively humane alternative choice while Rho A inhibitors remain hypothetical.

## Ethics Statement

The Chang Gung Human Investigation Committee approved all carried methods and all experimental protocols in this study, and the patients’ parents or guardians’ informed consent were obtained. We confirmed that all methods were performed in accordance with the relevant guidelines and standard regulations.

## Author Contributions

RL and W-IL designed the study and organized the team; W-IL, H-YW, Y-FL, and S-FT performed the immunologic assessments and genetic analysis; RL, M-WL, T-HJ, and J-LH took care of the patients; and R-CW studied the pathology. All of the authors contributed to this work and approved the manuscript.

## Conflict of Interest Statement

The authors declare that the research was conducted in the absence of any commercial or financial relationships that could be construed as a potential conflict of interest.

## Abbreviations

CID, combined T and B cell immunodeficiency; HSCT, hematopoietic stem cell transplantation; IBD, inflammatory bowel disease; ITK, IL-2-inducible T cell kinase; MIA, multiple intestinal atresia; NADPH, nicotinamide adenine dinucleotide phosphate oxidase; NEMO, nuclear factor kappa B essential modulator; ORAI-1, ORAI calcium release-activated calcium modulator 1; PICAR, Primary Immunodeficiency Care And Research; PIDs, primary immunodeficiency diseases; RAPID, Resource of Asian Primary Immunodeficiency Diseases; STAT1 or STAT3, signal transducer and activator of transcription 1 or 3; TPN, total parenteral nutrition; TPR, tetratricopeptide repeat; *TTC7A, tetratricopeptide repeat domain 7A*; WGS, whole-genome sequencing; XIAP, X-linked inhibitor of apoptosis; ZAP70, ZETA-associated protein, 70-KD.
