# Supplementary material for: Novel Mutations of the Tetratricopeptide Repeat Domain 7A Gene and Phenotype/Genotype Comparison
Source: Front Immunol. 2017 Sep 7;8:1066. doi: 10.3389/fimmu.2017.01066 (PMC5594067; doi:10.3389/fimmu.2017.01066)
Supplement: Supplementary file 1 [file Table_1.doc]

Supplemental Table 1. Whole genome sequencing revealed unreported compound heterozygous mutations in the dbSNP138 database that may affect protein function

| Gene | Chr | Position | Type | Ref | Var | Ref count | Var count | AA change |
| --- | --- | --- | --- | --- | --- | --- | --- | --- |
| Notch2 | 1 | 120572547 | SNV | T | C | 14 | 11 | Asn46Ser |
| Notch2 | 1 | 120572572 | SNV | C | T | 11 | 12 | Glu38Lys |
| TTC7A | 2 | 47177540 | SNV | G | A | 13 | 12 | Glu75Lys |
| TTC7A | 2 | 47202114 47202115 | Deletion | CT | - | 9 | 7 | Leu174fs |
| ANKRD36C | 2 | 96525718 | Replacement | G | TT | 30 | 27 | Frame shift |
| ANKRD36C | 2 | 96617111 | SNV | G | A | 36 | 49 | Nonsense |
| POTEF | 2 | 130832292 | SNV | T | A | 5 | 6 | Tyr918Phe |
| POTEF | 2 | 130877752 | SNV | T | C | 8 | 7 | Ser113Gly |
| ANLN | 7 | 36462370 | SNV | C | T | 9 | 7 | Arg810Cys |
| ANLN | 7 | 36463470 | SNV | G | A | 22 | 20 | Glu841Lys |
| DNAH10 | 12 | 124341723 | SNV | C | T | 10 | 12 | Arg2069Cys |
| DNAH10 | 12 | 124403451 | SNV | A | C | 9 | 8 | Ile3703Leu |
| ZNF717 | 3 | 75786252 | SNV | G | T | 42 | 34 | Pro841His |
| ZNF717 | 3 | 75786256 | SNV | T | A | 48 | 29 | Nonsense |
| ZNF717 | 3 | 75786379 | SNV | T | C | 36 | 57 | Thr799Ala |
| ZNF717 | 3 | 75786417 | SNV | T | C | 45 | 60 | Tyr786Cys |
| ZNF717 | 3 | 75786438 | SNV | G | A | 43 | 57 | Thr779Ile |
| ZNF717 | 3 | 75786450 | SNV | G | A | 45 | 57 | Thr775Met |
| ZNF717 | 3 | 75787057 | SNV | G | A | 31 | 48 | His573Tyr |
| ZNF717 | 3 | 75787159 | SNV | C | T | 40 | 41 | Glu539Lys |
| ZNF717 | 3 | 75787186 | SNV | C | T | 26 | 42 | Ala530Thr |
| ZNF717 | 3 | 75787279 | SNV | A | T | 25 | 27 | Trp499Arg |
| ZNF717 | 3 | 75787288 | SNV | T | C | 23 | 25 | Ile496Val |
| ZNF717 | 3 | 75787304 75787305 | MNV | AC | TT | 29 | 16 | Arg490Gln |
| ZNF717 | 3 | 75787405 | SNV | C | T | 31 | 21 | Gly457Arg |
| ZNF717 | 3 | 75787464 | SNV | C | T | 36 | 22 | Arg437Lys |
| ZNF717 | 3 | 75787486 | SNV | C | T | 35 | 32 | Glu430Lys |
| ZNF717 | 3 | 75787646 75787647 | Deletion | AA | - | 28 | 23 | Frame shift |
| ZNF717 | 3 | 75787869 75787870 | MNV | CA | TG | 38 | 62 | Cys302His |
| ZNF717 | 3 | 75787876 | SNV | G | T | 40 | 62 | Leu300Ile |
| ZNF717 | 3 | 75787927 | SNV | A | G | 44 | 49 | Tyr283His |
| ZNF717 | 3 | 75788023 | SNV | C | T | 33 | 61 | Val251Ile |
| ZNF717 | 3 | 75788115 | SNV | G | A | 39 | 67 | Thr220Met |
| OR4C3 | 11 | 48346547 | SNV | C | T | 32 | 22 | Pro19Ser |
| OR4C3 | 11 | 48347142 | SNV | C | T | 19 | 18 | Thr217Met |
| OR4C3 | 11 | 48347144 | SNV | T | C | 20 | 18 | Tyr218His |
| OR4C3 | 11 | 48347358 | SNV | C | T | 24 | 18 | Thr289Ile |
| OR4C5 | 11 | 48387900 | SNV | G | A | 29 | 17 | Nonsense |
| OR4C5 | 11 | 48387918 48387919 | Replacement | TG | A | 24 | 14 | Frame shift |
| OR4C5 | 11 | 48387946 | Replacement | G | CAT | 22 | 15 | Frame shift |
| CDC27 | 17 | 45214614 | Deletion | A | - | 26 | 48 | Frame shift |
| CDC27 | 17 | 45214617 45214618 | Insertion | - | C | 27 | 48 | Frame shift |
| CDC27 | 17 | 45214633 | SNV | A | C | 27 | 43 | Tyr606Asp |
| CDC27 | 17 | 45214648 | SNV | G | C | 26 | 28 | Gln601Glu |
| CDC27 | 17 | 45214651 | SNV | T | G | 26 | 30 | Ile600Leu |
| CDC27 | 17 | 45214654 | SNV | C | T | 25 | 24 | Ala599Thr |
| CDC27 | 17 | 45214699 | SNV | T | C | 25 | 17 | Ser584Gly |
| CDC27 | 17 | 45219223 | SNV | A | G | 41 | 31 | Met522Thr |
| CDC27 | 17 | 45219226 | SNV | T | G | 41 | 47 | Tyr521Ser |
