# Supplementary material for: Novel Mutations of the Tetratricopeptide Repeat Domain 7A Gene and Phenotype/Genotype Comparison
Source: Front Immunol. 2017 Sep 7;8:1066. doi: 10.3389/fimmu.2017.01066 (PMC5594067; doi:10.3389/fimmu.2017.01066)
Supplement: Supplementary file 2 [file Table_2.doc]

Supplemental Table 2. The genotypes and phenotypes of 49 patients with TTC7A mutations

| Mutation point | Ethnics [Ref.] | Mutation type | | | Onset  Neonate | Phenotype | | | | | | Significant events | | | Others | Survival  [Mortality cause] (alive age) |
| --- | --- | --- | --- | --- | --- | --- | --- | --- | --- | --- | --- | --- | --- | --- | --- | --- |
|  |  | Exon | type | TPR |  | MIA | Surgery | IBD | CID | | | Sepsis | HSCT | GvHD |  |  |
|  |  |  |  |  |  |  |  |  | T | B | NK |  |  |  |  |  |
| 223 G>A Glu75Lys  c. 520-521 del CT fs174X27 | Taiwan [P1 and P2 in this study] | 2  4 | Missense  Deletion | －  ＋ | Prenatal | ＋ | ＋ | ＋ |  | ＋ |  | ＋ |  |  |  | 8M [sepsis] |
|  |  |  |  | －  ＋ | Prenatal | ＋ | ＋ | ＋ |  | ＋ |  | ＋ |  |  |  | 4M [liver failure] |
| 1001+3 delAAGT, Del E7 | French-Canadian [33] | 7  Homo | Deletion | ＋ | ＋ | ＋ |  |  |  |  |  |  |  |  | Omphalocele | 3D [MIA] |
| 1001+3 delAAGT, Del E7 | French-Canadian [33] | 7  Homo | Deletion | ＋ | ＋ | ＋ |  |  |  |  |  |  |  |  |  | 2D [MIA] |
| 1001+3 delAAGT, Del E7 | French-Canadian [33] | 7  Homo | Deletion | ＋ | Prenatal | ＋ |  |  |  |  |  |  |  |  |  | Abortion [MIA] |
| 1001+3 delAAGT, Del E7 | French-Canadian [33] | 7  Homo | Deletion | ＋ | Prenatal | ＋ |  |  |  |  |  |  |  |  |  | 7D [MIA] |
| 1001+3 delAAGT, Del E7  c.2468T>C; p.L832P | French-Canadian/  English [33] | 7  20 | Deletion  Missense | ＋  ＋ | Prenatal | ＋ | ＋ |  | ＋ |  |  |  |  |  |  | 47D [MIA] |
| 1001+3 delAAGT, Del E7  c.2468T>C; p.L832P | French-Canadian/  English [33] | 7  20 | Deletion  Missense | ＋  ＋ | Prenatal | ＋ | ＋ | ＋ | ＋ | ＋ |  | ＋ | ＋ |  |  | 1Y [MIA] |
| c.1652 C>A; p.A551D  c.2482C>T; p.E828X | Irish/Ashkenazi Jewish [38] | 15  20 | Missense  Nonsense | ＋  ＋ | Prenatal | ＋ | ＋ |  | ＋ | ＋ | ＋ | ＋ |  |  | Microcephaly, hypotonia | Alive (7M) |
| c.2018-2 A>G; Del E18  c.2569G>T; p.E857X | Malaya [39] | 18  20 | Splicing  Nonsense | ＋  － | Prenatal | ＋ | ＋ |  |  |  |  | ＋ |  |  |  | 27M [sepsis] |
| c.2018-2 A>G; Del E18  c.2569G>T; p.E857X | Malaya [39] | 18  20 | Splicing  Nonsense | ＋  － | Prenatal | ＋ | ＋ |  |  |  |  | ＋ |  |  |  | 3D[sepsis] |
| c.1919+1G>A; Del E16 | Arabic [33] | 16  Homo | Splicing | ＋ | ＋ | ＋ | ＋ |  | ＋ | ＋ |  | ＋ |  |  | Enterococcus faecalis, Klebsiella | 3M [sepsis] |
| c. 313 Del TATC, Del E2, E3 | Serbian [37] | 2 | Deletion | ＋ | ＋ | ＋ |  |  |  |  |  |  |  |  |  | 1M [MIA] |
| c. 313 Del TATC, Del E2, E3 | Serbian [37] | 2 | Deletion | ＋ | ＋ | ＋ | ＋ |  |  |  |  |  |  |  | Line-Staphy aureus sepsis | 4M [sepsis] |
| c. 313 Del TATC, Del E2 E3 | Bosniak [37] | 2  Homo | Deletion | ＋ | ＋ | ＋ | ＋ |  | ＋ | ＋ |  | ＋ |  |  | Meconium peritonitis, pseudomonas aeruginosa, candida albicans, intraabdominal abscess | Alive (2.8Y) |
| c.762 Del AG | ? [37] | 5  Homo | Deletion | ＋ | ＋ | ＋ | ＋ |  | ＋ | ＋ |  | ＋ |  |  | Lines, UTI, G-tube, varicella infection | Alive (2Y) |
| c.1817A>G, pK606R c.2014T>C; pS672P | French-Canadian/mixed European [37] | 16  17 | Missense  Missense | －  － | Prenatal | ＋ | ＋ |  | ＋ | ＋ |  | ＋ | ＋ | － | E coli, line-sepsis, cirrhosis, lung dysfunction | Alive (22M) |
| c.2003C>A;p.S678X; c.2134C>T; p.Q712X | Italian [37] | 16  18 | Nonsense Nonsense | ＋  ＋ | ＋ | ＋ | ＋ | ＋ | ＋ | ＋ | ＋ |  | ＋ | ＋ | CMV pneumonitis | 10M [pneumonitis] |
| c.1196T>C; p.L399P | Italian [37] | 9  Homo | Missense |  | ＋ | ＋ | ＋ |  | ＋ | ＋ |  | ＋ |  |  | Klebsiella, E coli, Staphy. Aureus, candida sepsis, neurodevelopmental delay, failure to thrive, absence of visual evoked response | Alive (9M) |
| cDNA lack exon 2 and exon 3 | Italian [37] | 2  Homo | Deletion | ＋ | ＋ | ＋ | ＋ |  | ＋ | ＋ | ＋ | ＋ |  |  | Hepatosplenomegaly, failure to thrive | 8M [candida sepsis] |
| c.211G>A, p E71K | Paris [52] | 2  Homo | Missense | － | ＋ |  |  | ＋ | ＋ |  | ＋ |  |  |  |  | Alive (5M) |
|  |  |  |  | － | ＋ |  |  | ＋ | ＋ |  | ＋ |  | ＋ |  |  | 6M [HSCT] |
|  |  |  |  | － | ＋ |  |  | ＋ | ＋ | ＋ | ＋ |  | ＋ |  |  | 9M [HSCT] |
|  |  |  |  | － | ＋ |  |  | ＋ | ＋ |  | ＋ |  |  |  |  | 8M [enteropathy] |
|  |  |  |  | － | ＋ |  |  | ＋ | ＋ |  | ＋ |  |  |  |  | Alive (10M) |
|  |  |  |  | － | ＋ |  |  | ＋ | ＋ |  | ＋ |  |  |  |  | Alive (10M) |
|  |  |  |  | **－** | ＋ |  |  | ＋ | ＋ |  | ＋ |  |  |  |  | Alive (2Y) |
|  |  |  |  | － | ＋ |  |  | ＋ | ＋ | ＋ | ＋ |  |  |  | Alopecia, nail fungus | Alive (4Y) |
|  |  |  |  | － | ＋ |  |  | ＋ | ＋ | ＋ | ＋ | ＋ |  |  |  | 4Y [sepsis] |
|  |  |  |  | － | ＋ |  |  | ＋ | ＋ |  | ＋ |  |  |  | Alopecia, autoimmune hepatitis, nail fungus | 14Y [Gastric Carcinoma] |
|  |  |  |  | － | ＋ |  |  | ＋ | ＋ | ＋ | ＋ |  |  |  | Alopecia, autoimmune hemolysis | Alive (14Y) |
|  |  |  |  | － | ＋ |  |  | ＋ | ＋ |  | ＋ |  |  |  | Alopecia, IDDN, psoriasis, nail fungus | Alive (28Y) |
|  |  |  |  | **－** | ＋ |  |  | ＋ | ＋ | ＋ | ＋ |  |  |  | Bronchiectasis, alopecia, thyroiditis, nail fungus | Alive (50Y) |
| Del911T p.L304 fs X59; c.1433T>C, p. L478P | Paris [52] | 7  12 | Deletion Missense | － | ＋ |  |  | ＋ |  |  |  |  |  |  | Sparse hair, loss eyebrow, alopecia | Alive (18M) |
| c.Del 185-348; Del pD62-S116  c.Del 185-517; Del pD62-173G | Mixed European [35] | 2  3 | Deletion  Deletion | － | ＋ | ＋ | ＋ |  | ＋ | ＋ |  |  |  |  | TPN-cirrhosis | 2.5Y [sepsis] |
| C 829 C>T ; p.Q277X | Saudi Arabia [35] | 6 Homo | Nonsense | ＋ | ＋ | ＋ | ＋ |  | ＋ | ＋ | ＋ | ＋ | ＋ | ＋ | TPN-cirrhosis, line-sepsis (Klebsiella), pseudomonas pneumonia sepsis | 9M [pulmonary hemorrhage] |
| Del c. 2496 CG, p832X | Sri Lanka [35] | 20  Homo | Deletion | ＋ | ＋ | ＋ | ＋ |  | ＋ | ＋ |  |  |  |  | TPN-cirrhosis, bronchiectasis, pachyderma | Alive (73M) |
| c.1288-1392 del;  c.1616C>T | Norway [35] | 14  14 | Deletion  Missense | ＋ | ＋ | ＋ | ＋ |  | ＋ | ＋ |  | ＋ |  |  | PJP pneumonitis, Klebsiella sepsis | 8M [sepsis] |
| c.1008C>G; p.Y336X  c.1479 delG; L493 fsX13 | Mixed European [35] | 8  12 | nonsense  deletion | ＋ | ＋ | ＋ | ＋ |  | ＋ | ＋ |  |  |  |  |  | 3.9Y [virus pneumonia] |
| c.1510+105 T>A;  c.1673 ins G | Mixed European [35] | 12  15 | Splicing  Insertion | ＋ | Prenatal | ＋ | ＋ |  | ＋ | ＋ |  |  |  |  | Heart failure by mitral stenosis | 7M [CMV pneumonitis] |
| c.211G>A; p.E71K; c1576C>T;p.Q526X | Caucasian/  Sudanese [36, 42] | 2  15 | Missense  Nonsense | ＋ | ＋ | ＋ | ＋ | ＋ | ＋ | ＋ |  | ＋ |  |  | Strep. viridans, CONS, serratia sepsis | 11M [parainfluenza pneumonia, ARDS] |
| c.844-1G>T, losing exon 7  c.1204-2A>G, losing exon 10 | Caucasian [36] | 7  10 | Splicing  Splicing | ＋ | Prenatal | ＋ | ＋ |  | ＋ | ＋ |  |  |  |  |  | 3M [pulmonary emboli] |
|  |  |  |  |  | ＋ | ＋ | ＋ |  | ＋ |  |  |  |  |  |  | 19M [cardiac arrest] |
| c.2494 G>A; pA832T | Caucasian [36] | 20  Homo | Missense | ＋ | ＋ |  |  | ＋ |  |  |  |  |  |  | Failure to thrive | Alive (14M) |
|  |  |  |  |  | ＋ |  |  | ＋ |  |  |  |  |  |  | Failure to thrive | 11M [candida sepsis] |
| c.1037 T>C; pL346P | Turkish [40] | 8  Homo | Missense | － | ＋ |  |  | ＋ | ＋ | ＋ |  | ＋ |  |  | Klebsiella pneumonia and sepsis, pyelonephritis, | 15M [sepsis] |
| p.Glu191Fs;  p.I854Phe | Mediterranean [41] | 4  20 | Deletion  Missense | ＋  － | ＋ | ＋ |  |  |  |  |  | ＋ | ＋ |  | hypothyroidism sensory hearing loss, liver cirrhosis | Alive (77M) |
| p.Gly45-Ala55Del | Middle East [41] | 1  Homo | Deletion | ＋ | ＋ | ＋ |  |  | ＋ | ＋ |  | ＋ | ＋ |  | Langerhans cell histiocytosis in skin and skull [11M], recurrent E coli sepsis [post-HSCT], | Alive (86M) |
| p.Glu71Lys  p.Glu96 | British [41] | 2  2 | Missense  Missense | －  ＋ | ＋ | ＋ |  |  | ＋ | ＋ |  |  | ＋ |  | Line-sepsis, Rituximab for EBV (no ALPS), gastrostomy for diet | Alive (20M) |
